# Research Trends and Hot Spots in Telemedicine for the Elderly: A Scientometric Analysis

**DOI:** 10.3390/healthcare12181853

**Published:** 2024-09-14

**Authors:** Huiqian He, Salwa Hanim Abdul-Rashid, Raja Ariffin Raja Ghazilla

**Affiliations:** 1 Centre for Sustainable and Smart Manufacturing, Department of Mechanical Engineering, Faculty of Engineering, Universiti Malaya, Kuala Lumpur 50603, Malaysia; s2122009@siswa.um.edu.my; 2 School of Art Design, Guangdong Technology College, Zhaoqing 526100, China

**Keywords:** telemedicine, older adults, technology acceptance, bibliometrics, CiteSpace

## Abstract

Background: As the elderly population rapidly grows, age-related health issues are increasing. Telemedicine helps older adults adapt by providing efficient and accessible health management and medical services. Objectives: This study employs bibliometric analysis to examine research focus areas, emerging trends, and collaboration networks in telemedicine for older adults over the past three decades. Methods: The Web of Science Core Collection served as the primary data source for the publications on telemedicine and the elderly since the database’s inception through June 2024. Using CiteSpace.6.2.R4 software, keyword and collaboration network visualizations were generated, including clusters, co-authors, and co-citations. Results: This study analyzed 586 papers from 252 countries or regions, which were published across 246 journals and written by 2750 authors. Conclusions: The analysis revealed three primary research directions encompassing 42 clusters: (1) health literacy and technology adaptation; (2) telemedicine technology and health management; and (3) social interaction and economic impact. Research hotspots include elderly fitness, mobile health, technology acceptance, telemedicine, elderly care, and health literacy. Despite the potential benefit of telemedicine, challenges persist in areas such as technology acceptance, usability, effectiveness, service quality, and privacy concerns. This review provides a comprehensive overview of current research on telemedicine for the elderly and highlights emerging trends in the field.

## 1. Introduction

The global elderly population is rapidly expanding and is projected to double from 1.1 billion in 2021 to nearly 2.1 billion in 2050, representing 21% of the world’s total population [1]. As people age, they often experience a decline in mental acuity, physical coordination, physiological health, memory, and attention [2]. This large and growing elderly population represents a significant potential market for telemedicine services. Telemedicine for the elderly involves using communication technologies to provide medical diagnosis, health monitoring, health management, and other services, facilitating effective doctor-patient interactions [3]. The development of telemedicine for the elderly is crucial not only for their quality of life, health, and well-being but also has significant implications for service model innovation, resource allocation, and healthcare policies. However, the application of telemedicine in the elderly population still faces numerous challenges, such as technical barriers, service quality, data security, and privacy protection [4]. To promote health equity and sustainable elderly healthcare, it is essential to understand current trends and hot spots in telemedicine research.

Bibliometric analysis is a powerful tool for understanding the current state and future directions of telemedicine research for the elderly population. By applying mathematical and statistical methods to quantitatively analyze publications, bibliometrics can uncover the underlying knowledge structure in the scientific literature based on features such as the number of publications, authors, and journals [5]. It can identify research hotspots, emerging frontiers, and predicts the future developmental direction of telemedicine research [6]. Scientometric analysis, in addition to examining publications, also offers a historical and dynamic overview of a field, forming network and hierarchical summaries, detecting trends, gaps, limitations, impacts, and biases, thereby aiding informed decision-making [7]. Several recent studies have demonstrated the value of bibliometric analysis in exploring various aspects of telemedicine for the elderly. For example, Li [8] used bibliometrics to investigate design methods for the elderly in 1221 documents, while Yuan [6] employed CiteSpace software to analyze the literature on remote care for elderly patients with chronic diseases from 2002 to 2022. Similarly, Wang [5] examined the research status, collaborative network, and research hotspots of artificial intelligence in elder care over the past 23 years. Bibliometric studies also revealed important gaps and ethical considerations in telemedicine research. Waqas [9] noted a shift of teleradiology to mental health, stroke, and critical care from 2010 to 2019 but identified a lack of research on telemedicine financing, e-health literacy, and skills development. They also highlighted the need for clearer ethical standards, treatment protocols, and guidelines in the field. Similarly, Lwin [10] observed that the e-health literature rarely addresses patient confidentiality and ethical issues despite their critical importance in providing safe and equitable remote healthcare services. Looking to the future, Dai [11] investigated healthcare applications that used Multi-Criteria Decision Analysis (MCDA) techniques such as TOPSIS and Fuzzy AHP from 1999 to 2021, suggesting that big data and telemedicine will be key trends in the coming years.

Despite the existence of bibliometric analyses, systematic reviews, and meta-analyses, there remains a need for a comprehensive and in-depth large-scale analysis of the research hotspots and development trends in telemedicine for the elderly population. To address this gap, we conducted a scientometric analysis to create a systematic evolutionary map of the field. This approach allowed us to thoroughly analyze research related to telemedicine for the elderly, summarize the current research status, identify hotspots and trends, and determine research network characteristics and abundance. By filling this research gap, this study aims to provide a valuable reference and guide for future researchers in this area. The main research questions addressed in this paper are as follows:

1. What are the current research status and hot spots of telemedicine for the elderly?
2. What are the emerging research trends in telemedicine for the elderly?
3. What are the limitations and gaps in current research on telemedicine for older adults?
4. Which countries and regions are placing significant emphasis on research in telemedicine for the elderly?

## 2. Research Methodology

**Figure 1:**
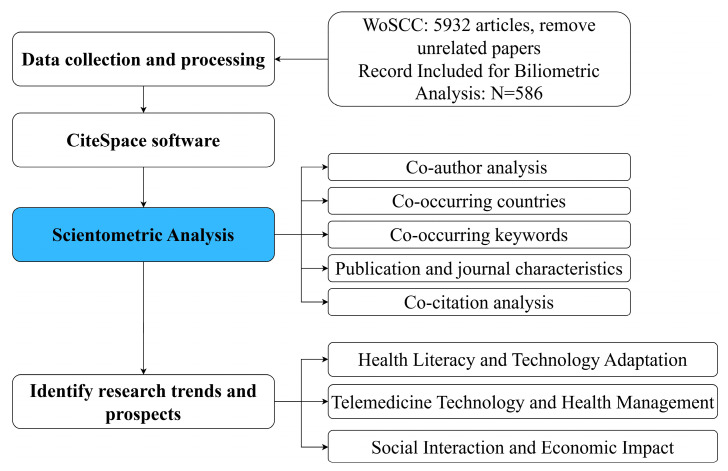
The literature screening and research framework process.

This paper provides a comprehensive review of telemedicine for the elderly through scientometric analysis. Figure 1 illustrates the data collection and processing procedure employed in this study. A scientometric analysis is used to objectively map the field of scientific knowledge and contribute to an in-depth understanding of the research status. The visualization of keywords and collaboration networks is performed using CiteSpace.6.2. R4 software.

### 2.1. Data Collection and Processing

To meet the specific requirements of bibliometrics and enhance the representativeness and accessibility of data [12], the analysis is based on data from the Web of Science Core Collection (WoSCC) Database, which includes SCI-EXPANDED, SSCI, A&HCI, and ESCI [11]. The data covers the period from 1 January 1989 to 13 June 2024. Given the earlier onset of diseases and the age criteria in telemedicine studies, this review defines the elderly as 50 years and above.

Telemedicine or telehealth refers to the use of information and communication technologies to exchange information during the diagnosis and treatment of diseases, as well as its research and evaluation [13]. Telemedicine, mobile health, eHealth, digital health, and smart health all focus on providing healthcare services remotely, and there are many overlapping relationships between them [14]. Considering the strong relationship, these terms were included in the retrieval framework.

To ensure the authenticity and comprehensiveness of the data sample, after multiple search experiments, the topic (TS) field was used for data retrieval, and the search string was set to the following: (TS = ((“the elderly” OR “elderly” OR “elderly people” OR “senior citizens” OR “seniors” OR “aged” OR “older adults”) AND (“telemedicine” OR “telehealth” OR “mHealth” OR “eHealth” OR “digital health” OR “smart healthcare”))). The search time range was set to all years, the language was restricted to English, and only the document types “article” and “review article” were included in the study, yielding 5932 articles from WoSCC. The search date was 13 June 2024. To ensure the accuracy of the study, the following were excluded: (1) conference abstracts and book chapters, (2) reports and design work, (3) personal interview reports, conference news, newspapers, conference speeches, and other articles, and (4) incomplete literature and articles irrelevant to the research topic. While the search results may not include all articles on elderly telemedicine, the criteria used identified high-quality articles that effectively represent its trends and status in the medical industry. Figure 1 illustrates the literature screening and research framework process. 

### 2.2. Scientometric Analysis Methods

The scientometric analysis involves examining large-scale literature datasets to create knowledge structures and evolution diagrams, employing quantitative methods to help researchers systematically understand the development of the industry field [15]. In this study, we utilized CiteSpace 6.2.R4 software for the literature analysis. CiteSpace is a Java-based scientometric analysis and visualization software that focuses on revealing the underlying knowledge in the scientific literature. The software efficiently processes and visualizes literature data [9] through various analyses, including keyword co-occurrence, co-citation, time series analysis, trend, and hot spot visualization, ultimately guiding researchers in identifying emerging topics and providing knowledge structure information and potential research directions [6].

The saliency measure in CiteSpace is a comprehensive tool that combines structural measures, time measures, and sigma measures to evaluate the importance of nodes, which can represent authors, countries, or publications. The graphs generated in CiteSpace use two quantified metrics to identify key nodes: betweenness centrality and burst strength [15]. Betweenness centrality is based on the degree to which a node lies on the shortest path between others, thereby potentially controlling communication [16]. A high centrality score suggests that the node has strong connectivity between unrelated clusters [17]. Additionally, burst detection analysis is employed to identify abrupt changes in citation frequencies within a specific timeframe, utilizing Kleinberg’s algorithm [18]. This analysis helps to highlight emerging trends and significant developments in a research field. The sigma score of a node combines its centrality and burstiness to quantify scientific novelty, while the Pathfinder method enhances network readability by removing redundant links between nodes [19]. 

Modularity (Q score) and Silhouette (S score) are two essential metrics used to evaluate the quality of clustering and the effectiveness of network decomposition [17]. The Q score is a global metric that assesses the network’s structure using clustering or community detection methods. It quantifies the strength of the network’s division into clusters or modules by tracking changes in Modularity values in response to new information [7]. The Q score ranges from 0 to 1, with values closer to 1 indicating a well-structured network with clearly defined clusters [17]. When the Q score falls below 0.30, it suggests that multiple clusters may be connected [19]. In a highly modular network, the connections between nodes within the same cluster are dense, while connections between nodes in different clusters are sparse [7]. On the other hand, the S score assesses the performance of the clustering algorithm by measuring the similarity between a sample within its cluster and samples in other clusters, thus evaluating the quality of clustering configurations [19]. The Silhouette value ranges from −1 to 1, with a value of 1 indicating that a sample is perfectly separated from other clusters [17]. Values close to 0 suggest that clusters may be overlapping, while negative values indicate that samples may have been misassigned to clusters [20]. When interpreting results using CiteSpace, it is essential to consider both the Modularity and Silhouette scores to ensure a valid and reasonable interpretation of the data [19].

Subsequently, three scientometric techniques were applied to analyze the data as follows: (1) Co-author analysis: this technique identifies a co-occurrence network of authors and countries, aiming to reflect collaboration at both micro and macro levels; (2) co-occurring keywords: this analysis consists of two parts—keywords timeline analysis and burst analysis; and (3) co-citation analysis: this technique includes the analysis of co-cited reference networks, co-cited authors, and co-cited journals. 

## 3. Results

**Figure 2:**
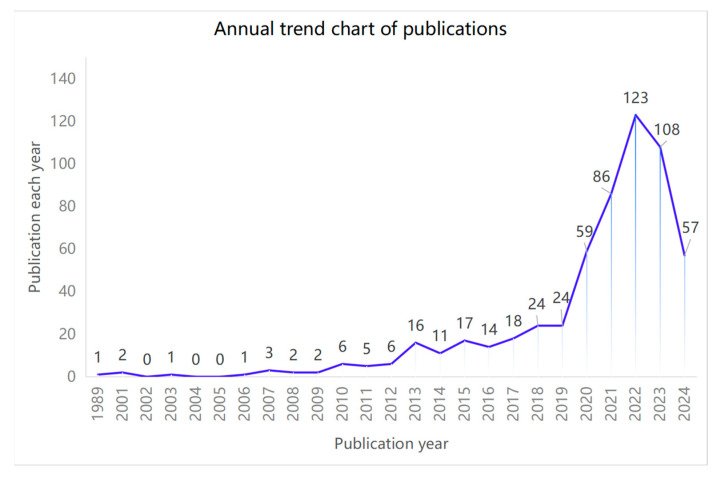
Annual related publications and trends (total 586).

The Web of Science search yielded a total of 5932 papers, which were then refined through data cleaning to include 586 papers in the final analysis. These papers originated from 252 countries or regions, were published across 246 journals, and were authored by 2750 individuals. Figure 2 illustrates the annual publication trends, revealing a gradual increase in the number of papers on telemedicine for the elderly since the first paper in 1989, reaching 24 publications in 2019. Notably, there was a sharp rise in publications from 2020 to 2022, accounting for 64.16% (376/586) of all publications, despite a slight decrease in 2023. This four-year period exhibits a peak point in research output, with a significant focus on public health, health technology, and electronic health literacy in 2022, largely influenced by the COVID-19 pandemic. The annual publication numbers and trends are visually represented in Figure 2, providing a clear overview of the research landscape in this field.

### 3.1. Co-Author Analysis

The advancement of information technology and increased academic exchanges have fostered research collaboration, resulting in the formation of complex networks that reflect academic cooperation and knowledge exchange. This section analyzes author collaboration patterns to gain insights into the scientific structure, identify key contributors in the field of telemedicine for the elderly, and explore their impact on academic progress. Network graphs will be used to depict collaborations between individuals and countries, providing a comprehensive understanding of the dynamics and impact of scientific collaboration from both micro and macro perspectives. The findings offer valuable insights for future research and policymaking.

#### 3.1.1. Co-Authorship Network

The top six authors with the highest number of journal publications in this field are Olivier Beauchet, Peter Anderberg, Namkee G. Choi, Jonathan Bayuo, Arkers Kwan Ching Wong, and Frances Kam Yuet Wong, each with four publications. Olivier Beauchet, Peter Anderberg, and Namkee G. Choi were from Canada, Sweden, and the USA, respectively. The latter three authors, all from China, have a strong collaborative relationship and have contributed to four joint articles among those analyzed.

**Figure 3:**
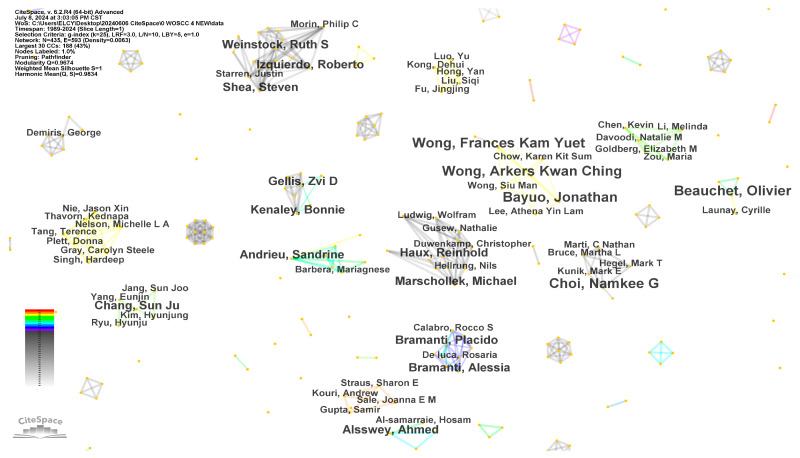
Co-authorship network.

In academia, journals and papers serve as static carriers of information without direct connections. The real drivers of academic exchanges and knowledge dissemination are the authors, who bridge different fields through their research publications. As such, the co-author network is essential for analyzing academic exchanges and collaboration. Figure 3 provides a visual representation of co-author cooperation, where nodes denote authors, and their size is proportional to their publication output. Larger nodes indicate higher academic output, while thicker edges signify closer collaboration. The network comprises 435 nodes and 593 edges, forming a complex collaboration network. The colors of the edges transition from gray to cooler tones and then to warmer tones, marking the years of collaboration from 1989 to 2024.

In social network analysis, the influence of researchers is closely tied to their connections within the network. CiteSpace employs a betweenness centrality index to gauge the impact of researchers in academic networks. Nodes with high betweenness centrality serve as bridges between different fields in academia, facilitating cross-disciplinary integration. The purple ring visualization tool in CiteSpace is used to display the betweenness centrality of nodes. A purple ring value exceeding 0.1 suggests that the node plays a significant intermediary role in the network, exerting a substantial influence on academic exchanges and knowledge dissemination [17]. However, in the networks depicted in Figure 3, the betweenness centrality of all nodes is 0, indicating that the connections between different research communities are not strong. This lack of close ties may hinder the flow of information and knowledge exchange among researchers. To address this issue and promote the free flow of knowledge and innovation, it is crucial to strengthen academic exchanges and cooperation in the future. 

#### 3.1.2. Co-Cited Countries

**Figure 4:**
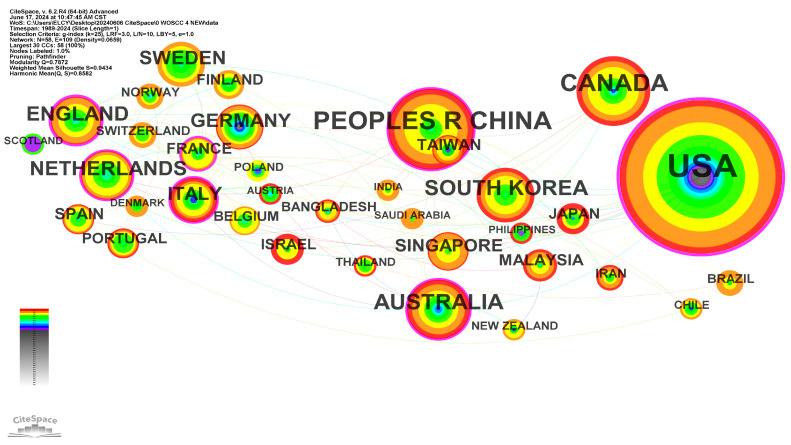
Co-authors’ countries network between the years 1989–2024.

The co-citation country network clusters demonstrate reliable Modularity (Q = 0.7872) and Silhouette (S = 0.9434) scores, indicating a well-structured network. The network consists of 58 nodes and 109 links, as depicted in Figure 4. The size of each node represents the total number of articles published in the corresponding country between 1989 and 2024. The top five countries with the highest number of co-citations are the USA (counts = 231), China (74, including 12 in Taiwan), Canada (52), Australia (36), and South Korea (32). This finding suggests that these countries have made significant contributions to research on telemedicine for the elderly and that the medical industry in these regions is well-advanced in this field. The USA exhibits the highest centrality (0.56), followed by England (0.34), the Netherlands (0.18), Australia (0.14), and France (0.13). These countries serve as key bridging nodes in the network and have considerable influence on cooperation in telemedicine research. They play a vital role in knowledge dissemination and information flow within the network. Setting the γ value to 0.5 reveals the top five countries with the strongest citation bursts, which are Germany (duration = 2007–2019, strength = 2.3), the USA (2008–2014, 15.12), Slovenia (2013–2016, 1.84), England (2018–2019, 2.2), and Sweden (2019–2021, 3.08). These citation bursts demonstrate significant advancement in elderly telemedicine research within these countries during the specific periods. 

### 3.2. Keyword Co-Occurrence and Evolution Analysis

**Figure 5:**
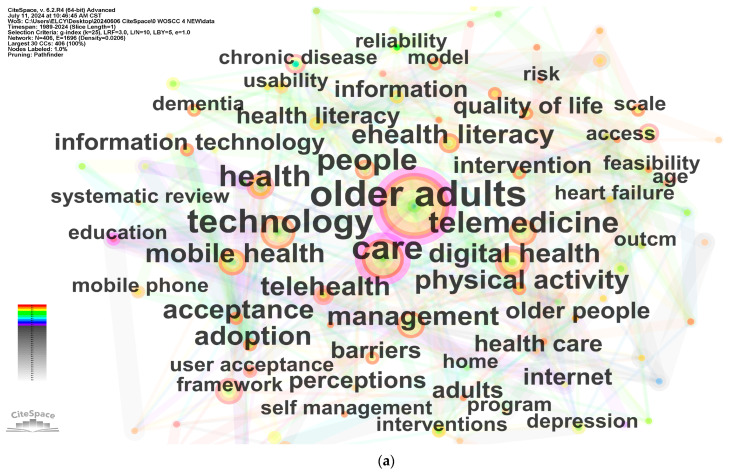

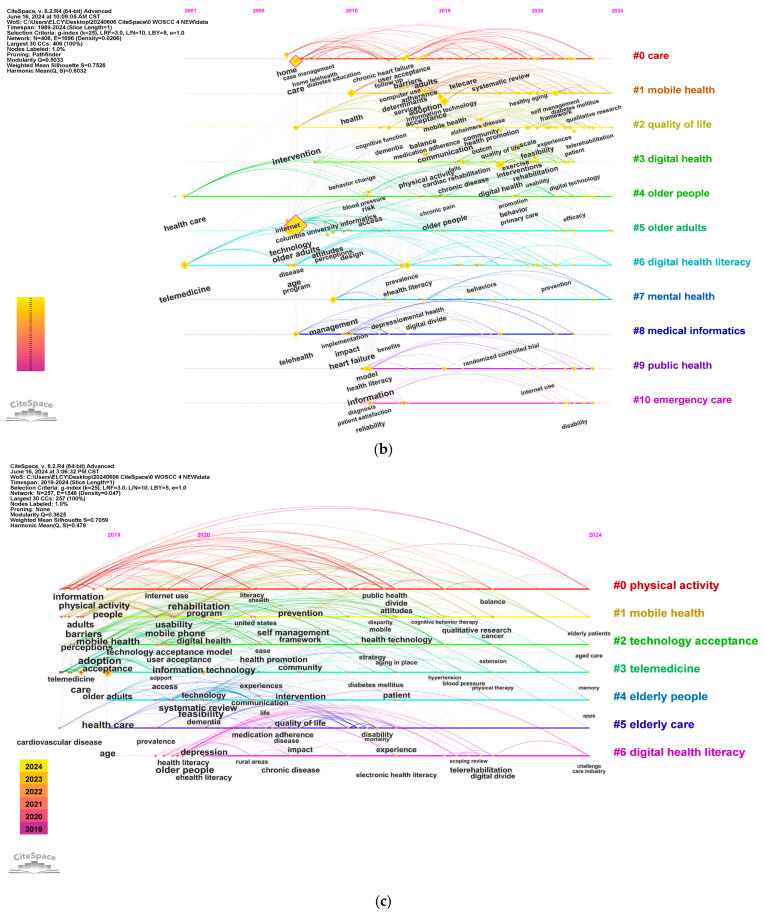
Keywords network and timeline analysis. Co-citation keyword networks are shown from 1989–2024 (**a**), timeline analysis from 1989–2024 (**b**) and 2019–2024 (**c**).

Keywords serve as concise representations of the core themes in research articles. CiteSpace’s keyword co-occurrence analysis reveals trending topics, while evolution networks illustrate the field’s development over time. Figure 5a depicts a network consisting of 406 nodes and 1696 links, and Figure 5b shows a network with 257 nodes and 1546 links. The size of the nodes in these networks represents the frequency of keywords in the bibliometric records. The number of nodes and connections in CiteSpace provides an intuitive reflection of the dynamics and knowledge structure of the research field, enabling researchers to quickly grasp research trends and relationships within the domain.

The co-occurrence analysis, as shown in Figure 5a, examines the frequency of keywords from 1989 to 2024. The top 10 most frequently used keywords, presented in the format “keyword (frequency count)”, are as follows: “older adults (243)”, “care (120)”, “technology (98)”, “telemedicine (62)”, “health (58)”, “people (56)”, “mobile health (52)”, “telehealth (50)”, “management (49)”, and “digital health (44)”. The timeline visualization in the figure is presented as a horizontal timeline, with clusters arranged from left to right and vertically in descending order of size [21]. The colored curves represent the co-citation links added in the corresponding year, and large nodes covered in red indicate citation bursts or highly cited cases [21]. The timeline analysis between 1989 and 2024 has a Modularity score of Q = 0.5033 (>0.3) and a Silhouette score of S = 0.7301 (>0.6), as shown in Figure 5b. The seven larger clusters identified between 2001 and 2024 are “care”, “mobile health”, “quality of life”, “digital health”, “older people”, “older adults”, and “digital health literacy”, with the largest cluster, Cluster 0, having 51 members.

To present the research hotspots in recent years more clearly, a keyword clustering co-occurrence network timeline analysis was also conducted on the literature from 2019 to 2024, as shown in Figure 5c. Clusters have different lifespans, with the largest cluster in 2019–2024, Cluster 0 “physical activity”, shown at the top of the view. This largest cluster has more than 48 members and is slightly less homogeneous than the smaller clusters in terms of Silhouette score. In this study, the review focuses mainly on seven clusters: “physical activity”, “mobile health”, “technology acceptance”, “telemedicine”, “elderly people”, “elderly care”, and “digital health literacy”. These clusters have acceptable Modularity (Q = 0.3625 > 0.3) and Silhouette (S = 0.7059 > 0.6) scores. Based on the frequency of occurrence, the top five keywords are older adults, care, technology, telemedicine, and health.

Burst detection is a powerful tool that identifies statistically significant fluctuations in citation frequency, allowing researchers to pinpoint sudden surges in reference citations [17]. The burst analysis conducted from 1989 to 2024 identified 11 keywords with a γ value of 0.7 that experienced notable bursts in citation frequency. These keywords are explained according to their burst strength, with the five highest being “outcome” (4.72), “home” (3.36), “telecare” (3.19), “services” (3), and “perceptions” (2.96). Focusing on the period from 2019 to 2020, the years with the most burst keywords at a γ value of 0.7, the top five keywords were “perceptions” (2.21), “telecare” (2.03), “services” (1.98), “clinical trials” (1.52), and “management” (1.52). This suggests that during this time, research interests were centered around understanding user perceptions, telecare services, and the management of clinical trials related to telemedicine for the elderly. Moving forward, from 2019 to 2021, “home” emerged as a significant burst keyword (2.16), indicating a growing focus on home-based telemedicine solutions for older adults. Between 2020 and 2021, “elderly people” (2.89) experienced a strong burst, highlighting the increasing attention given to this specific population in telemedicine research. Finally, from 2021 to 2022, the burst keywords “internet” (2.51) and “disease” (1.46) suggest a shift towards exploring the role of internet-based technologies in managing diseases among the elderly. When ranked by burst strength, “elderly people” (2.89) and “internet” (2.51) stand out as the keywords with the highest values. This shows the growing emphasis on home care services for the elderly since 2019, as researchers and practitioners recognize the potential of telemedicine in supporting aging in place and managing health conditions remotely. 

### 3.3. Co-Citation Analysis

Co-citation analysis is a valuable technique that assesses the frequency with which two articles are cited together in subsequent publications, providing insights into the relationships and similarities between documents [22]. This approach differs from co-occurrence analysis, which focuses on the frequency with which two variables appear together. Systematic mapping results generated by co-citation analysis include direct citation networks, co-citation, and co-occurrence networks, as well as clusters derived from these analyses [7]. CiteSpace.6.2.R4 software plays an important role in interpreting co-citation clusters and generating cluster labels through automatic cluster labeling [17]. This feature enables researchers to efficiently identify and understand the key themes and relationships within the analyzed literature. In this study, co-citation analysis is applied to three main elements: co-cited reference networks, co-cited authors, and co-cited journals.

#### 3.3.1. Publication and Journal Characteristics

*The Journal of Medical Internet Research* (publisher: JMIR Publications, established in 1999) emerged as the most cited journal, with 383 articles citing it between 1989 and 2024, with a centrality value of 0.03. *The Journal of Telemedicine and Telecare* (publisher: SAGE publishing, established in 1995) followed with 224 articles citing it. *The International Journal of Medical Informatics* (publisher: Elsevier Ireland Ltd., established in 1997) and *Telemedicine and e-Health* (publisher: Mary Ann Liebert Inc., established in 2000) were cited by 222 and 216, respectively. Table 1 presents the top 10 cited journals, demonstrating the high quality and significant impact of the articles published in them. 

**Figure 6:**
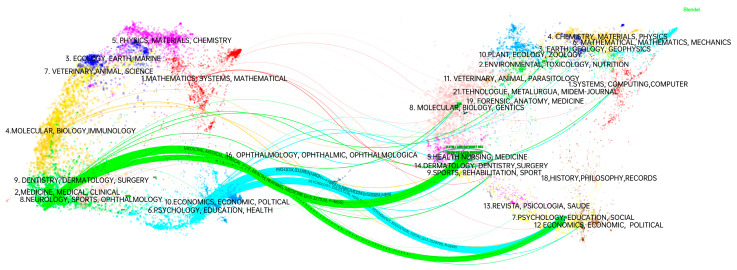
Journal double graph overlay analysis.

A research program or paradigm within a research field can be characterized by its knowledge base and research orientation [21]. To effectively compare and contrast the citation relationship between different journals, a journal double graph overlay analysis was conducted. This analysis reveals research hotspots and development trends from a macro perspective. Figure 6 illustrates the journal double graph overlay, with the citing journals on the left, the cited journals on the right, and the colored lines in the middle representing the citation relationships between them. The tags represent the journals covered by the topic [23]. After calculating the z-scores, the main paths are highlighted in bold. The journal double graph overlay belongs to the cross mode and passes through four main paths. First, “2. Medicine, Medical, Clinical” cites a large number of papers from “5. Health, Nursing, Medicine” (z = 5.327539, f = 4866, z = z-score, f = frequency of citations) and “6. Psychology, Education, Health” (z = 1.9342648, f = 1944). Second, “Psychology, Education, Health” cites a large number of papers from “5. Health, Nursing, Medicine” (z = 5.0801854, f = 4653) and “7. Psychology, Education, Social” (z = 1.9342648, f = 1944).

#### 3.3.2. Author Co-Citation Network

**Figure 7:**
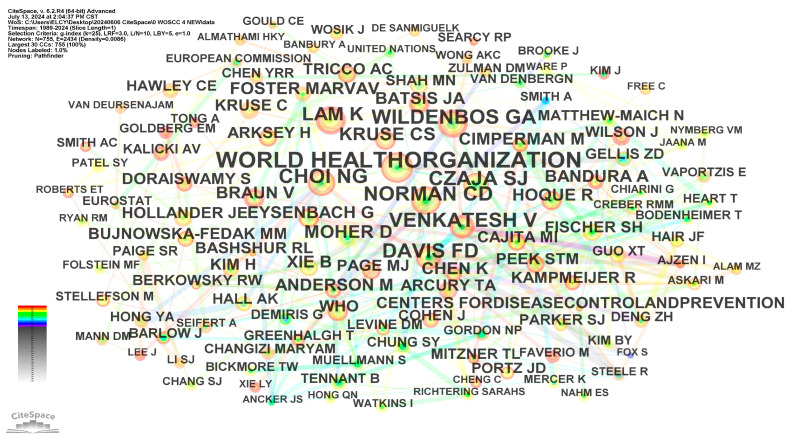
Author co-citation network.

Author co-citation analysis identifies and visualizes the intellectual structure of a research field by examining how frequently authors are cited together in the relevant literature [17]. Figure 7 presents the author’s co-citation network for the period 1989–2024, which consists of 755 nodes and 2434 links. In this network, nodes represent authors, while the connections between them signify co-citation relationships. The thickness or color of these connections indicates the strength or frequency of co-citation. Excluding anonymous authors, the most frequently cited individuals in the fields of telemedicine for the elderly are the World Health Organization (95 citations), Choi NG (64), Norman CD (62), Venkatesh V (60), Czaja SJ (59), Davis FD (56), Wildenbos GA (55), and Lam K (53). These authors have demonstrated significant influence and strong research relevance, establishing themselves as core researchers in the field of telemedicine for the elderly. Their contributions play a crucial role in advancing the knowledge base and shaping the development of telemedicine for the elderly.

In addition to the most frequently cited authors, burst strength analysis was employed to identify influential researchers with substantial citation bursts. The top eight authors with the strongest citation burst are Fox S (2009–2017, burst strength = 3.73), Or CKL (2013–2015, 3.72), Smith A (2015–2018, 5.81), Moher D (2017–2021, 7.34), Muellmann S (2018–2021, 4.59), Van DENBERGN (2020–2021, 3.7), Xie B (2021–2022, 3.75), and Hawley CE (2022–2024, 4.6). Among these authors, Moher D stands out with a remarkably high burst strength. In 2009, Moher D and his colleagues proposed the PRIMA method, which has become a landmark framework for reporting systematic reviews and meta-analyses.

#### 3.3.3. Document Co-Citation Analysis

**Figure 8:**
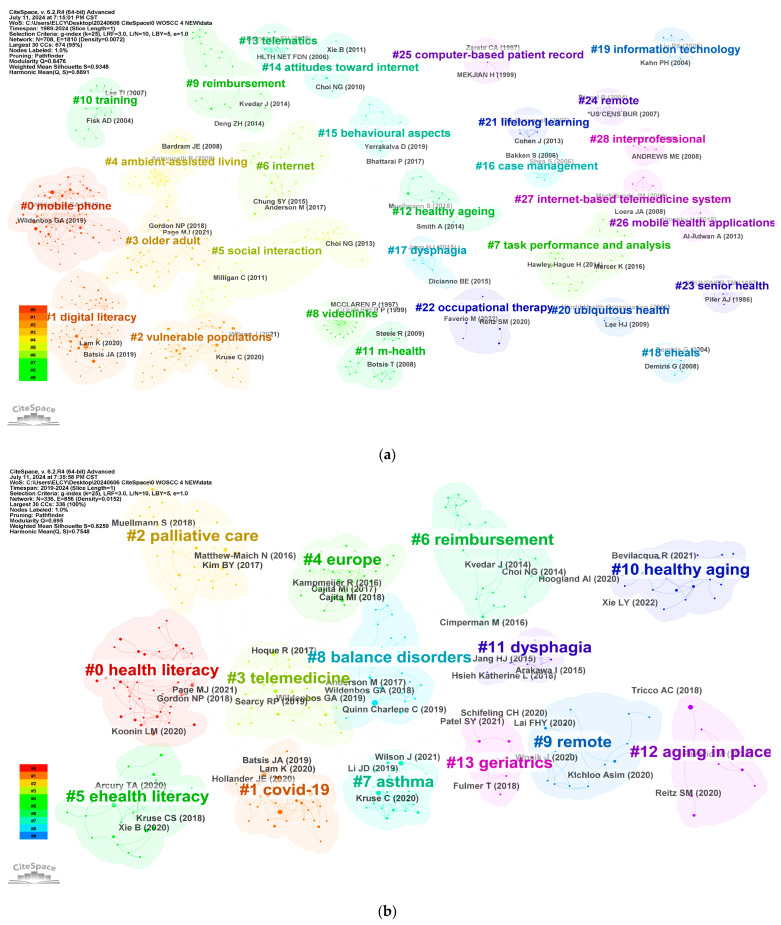
Co-citation reference network from the years 1989–2024 (**a**) and the years 2019–2024 (**b**).

Document co-citation analysis is a powerful method for revealing the intellectual structure and relationships within a field of study. By examining co-citation networks, researchers can uncover the nature of cited document clusters and their interconnections, providing insight into the underlying intellectual framework [17]. The co-cited reference graph from 1989 to 2024, shown in Figure 8a, revealed 42 distinct clusters with high Modularity (Q = 0.8476) and Silhouette (S = 0.9348) scores, indicating robust clustering. Reporting the cluster in the format of (year, size, Silhouette), the largest cluster, Cluster 0 (2017, 94, 0.83), has “mobile health; Alzheimer’s disease; design suggestions; human factor design; aging barriers” as keywords. This cluster is named “mobile phone”. This is followed by Cluster 1 (2019, 74, 0.879), with the keywords “digital health; health care access; mobile health; scoping review; coronavirus disease”, This cluster is named “digital literacy”. Cluster 2 (2020, 58, 0.868) has the keywords “COVID-19; telemedicine; geriatrics; Philadelphia; emergency medicine” and is named “vulnerable populations”. Cluster 3 (2018, 47, 0.826) has the keywords “digital health; virtual care; information technology; senior health; healthcare disparities” and is named “older adult”. These clusters illustrate the application and importance of mobile health in promoting digital literacy and improving the quality of life of vulnerable groups and the elderly. The top five highest citations are shown in Table 2.

To gain further insights into the latest trends in telemedicine for the elderly, we analyzed commonly cited references from 2019 to 2024 and their corresponding clusters, as depicted in Figure 8b. By examining the evolution of the network, it became evident that telemedicine garnered significant attention in 2019 and 2020, largely due to the COVID-19 pandemic. In recent years, researchers have increasingly focused on topics such as technology acceptance, e-health literacy, and medical interventions. The co-citation network diagram revealed 19 clusters with substantial Modularity (Q = 0.695) and Silhouette (S = 0.8259) scores. The four most prominent clusters were “health literacy”, “COVID-19”, “palliative care”, and “telemedicine”. The most frequently co-cited article, authored by Kenneth Lam et al. and cited 576 times, evaluated factors that may be overlooked when older adults transition to telehealth and assessed their readiness for video and telephone visits in various settings [24]. Based on these findings, the main research trends in recent years can be categorized into three main areas: (1) health literacy and technology adaptation, which involves improving older adults’ ability to obtain, understand, and apply health information, enhancing their communication and decision-making skills, self-management abilities, and overall adaptability and willingness to embrace telemedicine technology; (2) telemedicine technology and health management, encompassing the optimization of aging-friendly telemedicine designs, mobile health, video and information technology, and their application in health management and services for older individuals; and (3) social interaction and economic impact, which includes analyzing the performance of telemedicine in the elderly population, its influence on medical expenses reimbursement, and exploring how telemedicine can enhance the quality of life and social participation among older adults.

## 4. Discussion

This study aimed to investigate the research hotspots and trends in telemedicine for the elderly population. The analysis revealed a gradual increase in telemedicine literature from 1989 to 2024, with exponential growth observed between 2020 and 2023. Using CiteSpace software, the study examined the main keywords, co-citations, authors, journals, and countries in this research field. The most frequently cited keywords in the literature were “older adults”, followed by “care”, “technology”, and “telemedicine”. *The Journal of Medical Internet Research* had the highest number of publications, while the top five co-cited authors were the World Health Organization, Choi NG, Norman CD, Venkatesh V, and Czaja SJ. The United States demonstrated the highest centrality and co-citations, followed by China, which ranked second in the number of co-citations.

The co-cited reference network captured the evolution and relationships among 42 research clusters, revealing three main research trends, namely (1) health literacy and technology adaptation, (2) telemedicine technology and health management, and (3) social interaction and economic impact. These trends illustrate the multidimensional nature of telemedicine for the elderly, encompassing aspects such as telemedicine platform design, users’ health literacy, willingness to accept technology, social interaction, and economic changes. The findings indicate that researchers are highly concerned with the acceptance, adaptability and promotion of telemedicine among the elderly population.

### 4.1. Health Literacy and Technology Adaptation

Numerous factors influence digital health literacy, including computer characteristics, health knowledge, attitudes, education level, health status, health concern, marital status, Internet use duration and frequency, perceived ease of use, perceived reliability, perceived usefulness, computer pressure, age, and perceived risk [28,29]. Despite low digital health literacy and a lack of confidence and trust in obtaining and evaluating online medical resources, older adults are open to trying digital health. For instance, elderly individuals with cancer are willing to use home-based health information technology (HIT) [30]. Trust, negative attitudes, and technology anxiety are considered the primary barriers preventing users from adopting digital health platforms, and tailored educational activities may be able to address these issues [31]. The eHealth Literacy Scale, the most widely used tool for measuring digital literacy [32], assists medical professionals in assessing the e-health literacy of older adults, identifying vulnerable groups in digital health, and implementing personalized e-health interventions and education training [33].

Therefore, educational interventions are necessary to help older adults successfully use telehealth systems and improve their e-health literacy. Health literacy involves providing reliable and understandable health information to help individuals of all ages develop information evaluation and decision-making skills, enabling them to make appropriate choices [28,34]. Targeted efforts to increase telemedicine awareness among the elderly, integrate it with primary care, and ensure system ease of use [35] while providing older adults with information and communication technology (ICT) devices and internet access can help reduce health disparities and enhance the telehealth service delivery [36], potentially offering equitable healthcare access to rural and low-income populations [35].

Growing evidence suggests that telemedicine is a feasible and effective tool for managing chronic diseases and ensuring patient satisfaction among older adults. Studies by Şahin [37] and Rój [38] highlight the potential of telemedicine to deliver efficient health care and promote longevity in the elderly population [37,38]. Despite these benefits, telemedicine has yet to become a mainstream approach in healthcare delivery. To fully realize the potential of telemedicine, it must be regularly integrated into acute and emergency care settings, supported by flexible funding and staffing models that can transform traditional healthcare practices [39]. However, several challenges hinder the widespread adoption of telemedicine among older adults. The lack of face-to-face interaction and communication, inability to conduct physical examinations, digital literacy gaps, hearing difficulties, availability issues, and social concerns are significant barriers, as evidenced by multiple studies [4,37,40,41]. Furthermore, Hawley et al. (2020) identified interest, technology access, and confidence as major barriers to the uptake of home telehealth services [42].

Current telemedicine platforms often fail to adequately address the unique needs and challenges faced by older adults, including perceptual, cognitive, motivational, and physical limitations, as well as the lack of user-friendly interfaces and interactions. This observation is corroborated by the findings of Wildenbos [43] and Stefanicka-Wojtas and Kurpas [44]. To enhance the usability and accessibility of mobile health applications for elderly users, designers should consider factors such as visibility, coherence, navigation, data registration, privacy, fault tolerance, and interface visualization. Specific improvements may include increasing button and font sizes, enhancing color contrast, ensuring application consistency, simplifying application operations, and improving data visualization and security [45]. Addressing telehealth barriers, increasing seniors’ awareness of telehealth benefits, maximizing virtual interactions among seniors, and alleviating care imbalances are also crucial steps in improving adoption [4].

The low adoption and use of digital health among the elderly can be attributed to a complex interplay of factors. These include low social status, limited education, the rapid expansion of digital applications, broadband inequality, reduced familiarity, and structural problems [46,47]. The impact of age and digital maturity on technology acceptance and use has not been fully established, and research on digital health platforms lacks a unified theoretical framework [31]. A survey of older Americans revealed that while they are generally willing to use telemedicine, this willingness gradually declines with increasing age [48].

Numerous studies have demonstrated that the adoption of telehealth services by older adults is influenced by a complex interplay of factors, including context, environment, technology, economy, policy, and health status. Wu and Yu [49] highlighted the significant role of cultural background, medical literacy, and socioeconomic infrastructure in shaping older adults’ perceptions and use of telehealth and care services [49]. A wide range of factors, such as perceived usefulness, perceived needs, perceived ease of use, perceived benefits, familiarity, trust in technology, acceptance of suggestions, accommodations (family support), compatibility, financial costs, self-efficacy, satisfaction, learnability, memorability, performance expectations, effort expectations, and social influence, have been identified as key determinants of successful mobile health or e-health adoption among older adults. This finding is supported by substantial evidence from multiple studies [38,40,50,51]. However, several barriers can strongly inhibit the willingness of older adults to use telemedicine. These include the desire for autonomy, perceived risk, performance risk, privacy risk, and legal issues, followed by sunk costs, technological anxiety and transition costs. On the other hand, high availability and trust have been shown to have a significant positive impact on promoting the intention to use telemedicine, while inertia has a positive and significant impact on engagement [52,53,54,55]. To ensure the security of patient data, medical providers and stakeholders must prioritize data protection before accessing sensitive information. The lack of digitization of medical data also poses a significant challenge to the healthcare system [44]. Furthermore, the advancement of e-health requires the development of internet navigation skills, patient education, and collaborative efforts to improve the affordability and accessibility of applications [46].

### 4.2. Telemedicine and Health Management

Telemedicine has the potential to facilitate effective health management for older adults, but several challenges need to be addressed in the future. As telemedicine and mobile technology continue to advance, digital health platforms are becoming increasingly important tools for the elderly population. These platforms streamline data collection and help overcome barriers such as transportation difficulties, geographic limitations, limited clinic access, medical staff shortages, and inadequate community resources [56]. To ensure that telemedicine technologies meet the diverse and preference of older patients, it is crucial to adopt a user-centered approach in their design, testing, and evaluation [41,54]. This approach should address individual and systematic barriers to care, equity, and access [57]. The most successful mobile health interventions combine personalized feedback, interactive communication with healthcare professionals, and multifaceted features [58]. However, there is a lack of training for both patients and providers on the effective use of technology, as well as concerns about the cost of purchasing necessary equipment [41,59]. Mobile medical training has the potential to stimulate users’ cognitive abilities, including processing speed, foresight, episodic memory, and executive function [41,60]. When evaluating the usability of telemedicine, common methods include surveys, interviews, performance indicators, thinking aloud, behavioral observation, eye tracking, feedback logs, and screen recording [61]. However, the thinking-aloud method may affect the attention resources of elderly participants, preventing them from fully focusing on the evaluation of the technology and may not identify usability issues caused by physical impairments [43].

As the aging population grows, the incidence of chronic diseases such as cardiovascular disease, hypertension, and diabetes is increasing. Digital health technologies can effectively improve patient care and health outcomes, but strategic measures need to be taken to reduce the digital divide by considering the visual impairment, cognitive impairment, and dexterity of older adults [56]. Research has shown that mobile health interventions can improve glycated hemoglobin levels, self-management, self-care, and medication adherence in elderly patients with type 2 diabetes, thereby preventing future illnesses and enhancing overall health [58,59]. The current literature lacks detailed information on factors to consider when designing and developing mobile health solutions for type 2 diabetes self-management interventions, specifically for older adults [58]. Teleophthalmology has the potential to enhance access to specialists, reduce unnecessary visits and healthcare burdens, and save costs. However, evidence of its availability and effectiveness in older populations is limited [62]. Telemedicine has also been shown to support elderly heart failure patients by improving health equity, particularly for those in resource-poor areas or with physical disabilities, by overcoming transportation barriers and structural inequalities [47].

Currently, most research on telemedicine for the elderly focuses on treatment and rehabilitation care [54]. Research on the effectiveness of e-health, mobile health, and digital literacy in promoting fitness in older adults is still in its early stages, especially in terms of assessment tools and intervention methods [50,63]. There is also little evidence for the use of telemedicine in preventive and palliative care for the elderly population [54].

### 4.3. Social Interaction and Economic Impact

The adoption of telemedicine is significantly influenced by inequalities in broadband access and affordability of services. Structural and social determinants of health play a critical role in healthcare delivery and necessitate close collaboration among various stakeholders, including telecommunications companies, social service agencies, policymakers, and community organizations [47]. Older adults encounter age-related barriers to quality healthcare experiences, making them less likely to recommend technology to others. To address this, increased interaction and communication between healthcare practitioners, their patients and peers is essential [54].

The willingness of urban elderly individuals to use and pay for digital health technology is closely associated with factors such as age, exercise status, income, and medical history, with overall willingness being low. Policymakers should consider catering to the diverse needs of the elderly population, and medical insurance is expected to play a vital role in promoting the development of digital health [64]. When designing and implementing health behavior intervention programs for older adults, it is crucial to consider social capital and e-health literacy, as they serve as important mediating factors between structural social capital and health behaviors [65]. 

Multiple facilitators and barriers influence telehealth use among older adults. Interpersonal training may help reduce anxiety and improve literacy and acceptance [51], while data privacy issues pose challenges to the effective use of telemedicine and its underlying business value [66]. Telemedicine has shown mixed results in terms of cost-effectiveness, saving travel costs but also increasing expenses such as insurance [67]. Economic factors and cost are likely to impact the use of telemedicine services by the elderly, who tend to prefer more affordable e-health services [68]. It is recommended that medical insurance be incorporated into telemedicine in the future. In rural areas, expensive and unreliable network connections hinder the use of telemedicine by the elderly, with trust and choice being the primary barriers and facilitators. Offering telemedicine services through video can potentially increase utilization [69].

Considering the impact of social interaction and health behaviors of the elderly on community healthcare, it is necessary to develop infrastructure and provide training and support for patients and healthcare providers to effectively use telemedicine intervention technology while adhering to user-centered principles. Fanning [41] argued that telemedicine can enhance healthcare accessibility, but these technologies must meet the needs of the elderly, be patient-centered, and be perceived as usable and useful by older adults [41]. Furthermore, policymakers should strengthen clinical guidelines and policies, address privacy concerns, and work towards narrowing and bridging the digital divide [29,54] while also improving payment models [54]. Recommendations include stabilizing platforms, increasing supplier capacity, and garnering government policy support [70], as well as enhancing the medical welfare system for the elderly, optimizing the industrial structure of the elderly medical industry, adjusting hospital management models, strengthening market supervision and management, improving the consumer environment of the geriatric medicine market, forming interdisciplinary teams, and promoting the development of digital health tools and telemedicine systems, with a focus on higher safety, affordability, and accessibility [41,54,71].

## 5. Conclusions

This scientometric review provides the first comprehensive analysis of the development and trends in telemedicine for the elderly from a large-scale perspective. It reveals that research in this field began in 1989, with a rapid increase in publications from 2020 to 2023, largely influenced by the COVID-19 pandemic. The co-citation analysis identified “mobile phones” and “digital literacy” as the most prominent research clusters, indicating that telemedicine is primarily delivered through mobile devices and that digital health literacy among the elderly has become a key research focus. 

The review highlights three main research directions and trends: health literacy and technology adaptation, telemedicine and health management, and social interaction and economic impact. The most frequently cited keywords across all publications were “older adults” and “care”, with recent research focusing on physical activity, mobile health, technology acceptance, and telemedicine.

Geographically, the development of telemedicine for the elderly varies across countries. The co-author network is relatively dense in Europe, sparse in Asia, and minimal in Africa. The United States and China have the highest influence and total citations, while the United States and the United Kingdom exhibit the highest centrality. Sweden and Germany have shown significant growth in influence. However, the analysis reveals insufficient interdisciplinary collaboration among authors and limited collaboration between countries.

Despite the importance of telemedicine for elderly healthcare, no papers were found on systematically promoting and popularizing telemedicine among the elderly population. Additionally, many studies on elderly telemedicine technology acceptance included only small-scale samples and did not further investigate influencing factors or apply the results to real-world scenarios. Protecting user data and privacy remains a challenge, and improving digital health literacy among the elderly is an urgent issue. Future research should focus on providing telemedicine training and more personalized telehealth tools to meet the needs and expectations of older adults, addressing mental health concerns, improving social interaction, and including telemedicine medical insurance reimbursement.

The study has some limitations, including the exclusive use of the WoSCC database, which may not capture all relevant literature, the restriction to English-language publications, and the focus on first authors while not fully describing the contribution of other authors. In conclusion, the network analysis results of co-occurrence, co-cited, and co-citation in this review provide valuable insights for scholars in the field. Highly cited articles and journals with high publication volume can guide researchers in their reading and submission choices. This study helps researchers and decision-makers understand the current status, research hotspots, and collaboration networks in telemedicine for the elderly, facilitating informed decisions and improving work quality. The bibliometric analysis offers future research directions and suggestions for advancing the field of telemedicine for the elderly.

## Figures and Tables

**Table 1:** The top 10 cited journals (1989–2024).

Journal	Impact Factor (2022)	Count	Centrality	Number of Articles	Percentage of Articles (%)
*Journal of Medical Internet Research*	7.4	383	0.03	34	5.80%
*Journal of Telemedicine and Telecare*	4.7	224	0.04	18	3.07%
*International Journal of Medical Informatics*	4.9	222	0.05	18	3.07%
*Telemedicine and E-Health*	4.7	216	0.02	21	3.58%
*Journal of the American Geriatrics Society*	6.3	202	0.08	13	2.22%
*JMIR Mhealth and Uhealth*	5	190	0	15	2.56%
*International Journal of Environmental Research and Public Health*	4.614	150	0.01	18	3.07%
*PLoS ONE*	3.7	149	0.01	2	0.03%
*BMC Health Services Research*	2.8	138	0.02	6	0.10%
*Journal of the American Medical Informatics Association*	6.4	136	0.07	0	0

**Table 2:** The most co-cited references from 1989–2024.

Title	Author, Year	Count/Citations	Source	Vol, Page
Assessing Telemedicine Unreadiness among Older Adults in the United States during the COVID-19 Pandemic	Lam K, 2020 [24]	53/576	JAMA Intern Med	V180, P1389
Aging Barriers Influencing Mobile Health Usability for Older Adults: A Literature-Based Framework (MOLD-US)	Wildenbos GA, 2018 [25]	32/387	Int J Med Inform	V114, P66
Effectiveness of Ambulatory Telemedicine Care in Older Adults: A Systematic Review	Batsis JA, 2019 [26]	23/207	J Am Geriatr Soc	V67, P1737
Utilization Barriers and Medical Outcomes Commensurate with the Use of Telehealth among Older Adults: Systematic Review	Kruse C, 2020 [27]	23/199	JMIR Med Inf	V8, P0
Older Adult Internet Use and e-Health Literacy	Arcury TA, 2020 [28]	22/220	J Appl Gerontol	V39, P141

## Data Availability

Not applicable.

## References

[1] World Health Organization (2022). The UN Decade of Healthy Ageing 2021–2030 in a Climate-Changing World.

[2] Liu N., Yin J., Tan S.S.-L., Ngiam K.Y., Teo H.H. (2021). Mobile health applications for older adults: A systematic review of interface and persuasive feature design. J. Am. Med. Inform. Assoc..

[3] Vitali A., Ghidotti A., Savoldelli A., Bonometti F., Rizzi C., Bernocchi P., Borghi G., Scalvini S. (2023). Definition of a Method for the Evaluation of Telemedicine Platforms in the Italian Context. Telemed. E-Health.

[4] Haimi M., Goren U., Grossman Z. (2024). Barriers and challenges to telemedicine usage among the elderly population in Israel in light of the COVID-19 era: A qualitative study. Digit. Health.

[5] Wang J., Liang Y., Cao S., Cai P., Fan Y. (2023). Application of Artificial Intelligence in Geriatric Care: Bibliometric Analysis. J. Med. Internet Res..

[6] Yuan Y., Wang S., Tao C., Gu Z., Kitayama A., Yanagihara K., Liang J. (2024). Mapping trends and hotspots regarding the use of telenursing for elderly individuals with chronic diseases: A bibliometric analysis. Medicine.

[7] Amrapala A., Sabé M., Solmi M., Maes M. (2023). Neuropsychiatric disturbances in mild cognitive impairment: A scientometric analysis. Ageing Res. Rev..

[8] Li Y., Abdul-Rashid S.H., Ghazilla R.A.R. (2022). Design Methods for the Elderly in Web of Science, Scopus, and China National Knowledge Infrastructure Databases: A Scientometric Analysis in CiteSpace. Sustainability.

[9] Waqas A., Teoh S.H., Lapão L.V., Messina L.A., Correia J.C. (2020). Harnessing Telemedicine for the Provision of Health Care: Bibliometric and Scientometric Analysis. J. Med. Internet Res..

[10] Lwin H.N.N., Punnakitikashem P., Thananusak T. (2023). E-Health Research in Southeast Asia: A Bibliometric Review. Sustainability.

[11] Dai Z., Xu S., Wu X., Hu R., Li H., He H., Hu J., Liao X. (2022). Knowledge Mapping of Multicriteria Decision Analysis in Healthcare: A Bibliometric Analysis. Front. Public Health.

[12] Liu X., Zhao S., Tan L., Tan Y., Wang Y., Ye Z., Hou C., Xu Y., Liu S., Wang G. (2022). Frontier and hot topics in electrochemiluminescence sensing technology based on CiteSpace bibliometric analysis. Biosens. Bioelectron..

[13] Alexandra S., Handayani P.W., Azzahro F. (2021). Indonesian hospital telemedicine acceptance model: The influence of user behavior and technological dimensions. Heliyon.

[14] Wang Y. (2022). The Regulation of China Internet Health:the Analysis of Triple Logics and the Choice of Practice Strategies. Ph.D. Thesis.

[15] Zhong B., Wu H., Li H., Sepasgozar S., Luo H., He L. (2019). A scientometric analysis and critical review of construction related ontology research. Autom. Constr..

[16] Freeman L.C. (1977). A Set of Measures of Centrality Based on Betweenness. Sociometry.

[17] Chen C., Ibekwe-SanJuan F., Hou J. (2010). The structure and dynamics of cocitation clusters: A multiple-perspective cocitation analysis. J. Am. Soc. Inf. Sci. Technol..

[18] Kleinberg J. Bursty and hierarchical structure in streams. Proceedings of the Eighth ACM SIGKDD International Conference on Knowledge Discovery and Data Mining.

[19] Chen C. (2016). CiteSpace: A Practical Guide for Mapping Scientific Literature.

[20] Belyadi H., Haghighat A. (2021). Unsupervised machine learning: Clustering algorithms. Machine Learning Guide for Oil and Gas Using Python.

[21] Chen C. (2017). Science Mapping: A Systematic Review of the Literature. J. Data Inf. Sci..

[22] Zhou X., Zhou M., Huang D., Cui L. (2022). A probabilistic model for co-occurrence analysis in bibliometrics. J. Biomed. Inform..

[23] Pan Y., Deng X., Chen X., Lin M. (2023). Bibliometric analysis and visualization of research trends in total mesorectal excision in the past twenty years. Int. J. Surg..

[24] Lam K., Lu A.D., Shi Y., Covinsky K.E. (2020). Assessing telemedicine unreadiness among older adults in the United States during the COVID-19 pandemic. JAMA Intern. Med..

[25] Wildenbos G.A., Peute L., Jaspers M. (2018). Aging barriers influencing mobile health usability for older adults: A literature based framework (MOLD-US). Int. J. Med. Inform..

[26] Batsis J.A., DiMilia P.R., Seo L.M., Fortuna K.L., Kennedy M.A., Blunt H.B., Bagley P.J., Brooks J., Brooks E., Kim S.Y. (2019). Effectiveness of ambulatory telemedicine care in older adults: A systematic review. J. Am. Geriatr. Soc..

[27] Kruse C., Fohn J., Wilson N., Patlan E.N., Zipp S., Mileski M. (2020). Utilization barriers and medical outcomes commensurate with the use of telehealth among older adults: Systematic review. JMIR Med. Inform..

[28] Arcury T.A., Sandberg J.C., Melius K.P., Quandt S.A., Leng X., Latulipe C., Miller D.P., Smith D.A., Bertoni A.G. (2020). Older Adult Internet Use and eHealth Literacy. J. Appl. Gerontol..

[29] Liu S., Zhao H., Fu J., Kong D., Zhong Z., Hong Y., Tan J., Luo Y. (2022). Current status and influencing factors of digital health literacy among community-dwelling older adults in Southwest China: A cross-sectional study. BMC Public Health.

[30] Hoogland A.I., Mansfield J., Lafranchise E.A., Bulls H.W., Johnstone P.A., Jim H.S. (2020). eHealth literacy in older adults with cancer. J. Geriatr. Oncol..

[31] Frishammar J., Essén A., Bergström F., Ekman T. (2023). Digital health platforms for the elderly? Key adoption and usage barriers and ways to address them. Technol. Forecast. Soc. Chang..

[32] Oh S.S., Kim K.-A., Kim M., Oh J., Chu S.H., Choi J. (2021). Measurement of Digital Literacy Among Older Adults: Systematic Review. J. Med. Internet Res..

[33] Xie L., Hu H., Lin J., Mo P.K.H. (2024). Psychometric validation of the Chinese digital health literacy instrument among Chinese older adults who have internet use experience. Int. J. Older People Nurs..

[34] Nakayama K., Yonekura Y., Danya H., Hagiwara K. (2022). COVID-19 Preventive Behaviors and Health Literacy, Information Evaluation, and Decision-making Skills in Japanese Adults: Cross-sectional Survey Study. JMIR Form. Res..

[35] Fischer S.H., Ray K.N., Mehrotra A., Bloom E.L., Uscher-Pines L. (2020). Prevalence and Characteristics of Telehealth Utilization in the United States. JAMA Netw. Open.

[36] Choi N.G., DiNitto D.M., Marti C.N., Choi B.Y. (2022). Telehealth Use among Older Adults during COVID-19: Associations with Sociodemographic and Health Characteristics, Technology Device Ownership, and Technology Learning. J. Appl. Gerontol..

[37] Şahin E., Veizi B.G.Y., Naharci M.I. (2024). Telemedicine interventions for older adults: A systematic review. J. Telemed. Telecare.

[38] Rój J. (2022). What Determines the Acceptance and Use of eHealth by Older Adults in Poland?. Int. J. Environ. Res. Public Health.

[39] Smith A.C., Thomas E., Snoswell C.L., Haydon H., Mehrotra A., Clemensen J., Caffery L.J. (2020). Telehealth for global emergencies: Implications for coronavirus disease 2019 (COVID-19). J. Telemed. Telecare.

[40] Almulhem J.A. (2023). Factors, Barriers, and Recommendations Related to Mobile Health Acceptance among the Elderly in Saudi Arabia: A Qualitative Study. Healthcare.

[41] Fanning J., Brinkley T.E., Campbell L.M., Colon-Semenza C., Czaja S.J., Moore R.C., Pajewski N.M., Kritchevsky S. (2024). Research Centers Collaborative Network Workshop on Digital Health Approaches to Research in Aging. Innov. Aging.

[42] Hawley C.E., Genovese N., Owsiany M.T., Triantafylidis L.K., Moo L.R., Linsky A.M., Sullivan J.L., Paik J.M. (2020). Rapid integration of home telehealth visits amidst COVID-19: What do older adults need to succeed?. J. Am. Geriatr. Soc..

[43] Wildenbos G., Jaspers M., Schijven M., Peute L.D. (2019). Mobile health for older adult patients: Using an aging barriers framework to classify usability problems. Int. J. Med. Inform..

[44] Stefanicka-Wojtas D., Kurpas D. (2022). eHealth and mHealth in Chronic Diseases—Identification of Barriers, Existing Solutions, and Promoters Based on a Survey of EU Stakeholders Involved in Regions4PerMed (H2020). J. Pers. Med..

[45] Morey S.A., Stuck R.E., Chong A.W., Barg-Walkow L.H., Mitzner T.L., Rogers W.A. (2019). Mobile Health Apps: Improving Usability for Older Adult Users. Ergon. Des. Q. Hum. Factors Appl..

[46] Verma R., Saldanha C., Ellis U., Sattar S., Haase K.R. (2022). eHealth literacy among older adults living with cancer and their caregivers: A scoping review. J. Geriatr. Oncol..

[47] Creber R.M., Dodson J.A., Bidwell J., Breathett K., Lyles C., Still C.H., Ooi S.-Y., Yancy C., Kitsiou S. (2023). Telehealth and Health Equity in Older Adults with Heart Failure: A Scientific Statement from the American Heart Association. Circ. Cardiovasc. Qual. Outcomes.

[48] Odebunmi O., Hughes T.D., Waters A.R., Urick B.Y., Herron C., Wangen M., Rohweder C., Ferrari R.M., Marciniak M.W., Wheeler S.B. (2024). Findings from a National Survey of Older US Adults on Patient Willingness to Use Telehealth Services: Cross-Sectional Survey. J. Med. Internet Res..

[49] Wu Z., Yu P. (2021). The role of digital devices in chronic disease management during the COVID-19 pandemic—A study of senior citizens in Wuhan. Asian J. Commun..

[50] Wang X., Luan W. (2022). Research progress on digital health literacy of older adults: A scoping review. Front. Public Health.

[51] Lin T.T., Bautista J.R., Core R. (2020). Seniors and mobiles: A qualitative inquiry of mHealth adoption among Singapore seniors. Inform. Health Soc. Care.

[52] Vietzke J., Schenk L., Baer N.-R. (2023). Middle-aged and older adults’ acceptance of mobile nutrition and fitness tools: A qualitative typology. Digit. Health.

[53] Klaver N.S., van de Klundert J., Broek R.J.G.M.v.D., Askari M. (2021). Relationship Between Perceived Risks of Using mHealth Applications and the Intention to Use Them Among Older Adults in the Netherlands: Cross-sectional Study. JMIR Mhealth Uhealth.

[54] Zhang Y., Leuk J.S.-P., Teo W.-P. (2023). Domains, Feasibility, Effectiveness, Cost, and Acceptability of Telehealth in Aging Care: Scoping Review of Systematic Reviews. JMIR Aging.

[55] Tandon U., Ertz M. (2023). Shashi Continued Intention of mHealth Care Applications among the Elderly: An Enabler and Inhibitor Perspective. Int. J. Hum.–Comput. Interact..

[56] Krishnaswami A., Beavers C., Dorsch M.P., Dodson J.A., Masterson Creber R., Kitsiou S., Goyal P., Maurer M.S., Wenger N.K., Croy D.S. (2020). Gerotechnology for Older Adults with Cardiovascular Diseases *JACC* State-of-the-Art Review. J. Am. Coll. Cardiol..

[57] Wardlow L., Leff B., Biese K., Roberts C., Archbald-Pannone L., Ritchie C., DeCherrie L.V., Sikka N., Gillespie S.M. (2023). The Collaborative for Telehealth and Aging Development of telehealth principles and guidelines for older adults: A modified Delphi approach. J. Am. Geriatr. Soc..

[58] Sebastián J.V.-D., Ciudin A., Castellano-Tejedor C. (2021). Analysis of Effectiveness and Psychological Techniques Implemented in mHealth Solutions for Middle-Aged and Elderly Adults with Type 2 Diabetes: A Narrative Review of the Literature. J. Clin. Med..

[59] Kruse C.S., Mileski M., Heinemann K., Huynh H., Leafblad A., Moreno E. (2023). Analyzing the Effectiveness of mHealth to Manage Diabetes Mellitus Among Adults Over 50: A Systematic Literature Review. J. Multidiscip. Health.

[60] Maggio M.G., Luca A., Calabrò R.S., Drago F., Nicoletti A. (2024). Can mobile health apps with smartphones and tablets be the new frontier of cognitive rehabilitation in older individuals? A narrative review of a growing field. Neurol. Sci..

[61] Wang Q., Liu J., Zhou L., Tian J., Chen X., Zhang W., Wang H., Zhou W., Gao Y. (2022). Usability evaluation of mHealth apps for elderly individuals: A scoping review. BMC Med. Inform. Decis. Mak..

[62] Fatehi F., Jahedi F., Tay-Kearney M.-L., Kanagasingam Y. (2020). Teleophthalmology for the elderly population: A review of the literature. Int. J. Med. Inform..

[63] McGarrigle L., Todd C. (2020). Promotion of Physical Activity in Older People Using mHealth and eHealth Technologies: Rapid Review of Reviews. J. Med. Internet Res..

[64] Yang K., Li Y., Qi H. (2023). Determinants of and Willingness to Use and Pay for Digital Health Technologies Among the Urban Elderly in Hangzhou, China. Risk Manag. Health Policy.

[65] Cui G.-H., Li S.-J., Yin Y.-T., Chen L.-J., Li J.-Q., Liang F.-Y., Liu X.-Y., Chen L. (2021). The relationship among social capital, eHealth literacy and health behaviours in Chinese elderly people: A cross-sectional study. BMC Public Health.

[66] Pool J., Akhlaghpour S., Fatehi F., Gray L.C. (2022). Data privacy concerns and use of telehealth in the aged care context: An integrative review and research agenda. Int. J. Med. Inform..

[67] Rush K.L., Singh S., Seaton C.L., Burton L., Li E., Jones C., Davis J.C., Hasan K., Kern B., Janke R. (2022). Telehealth Use for Enhancing the Health of Rural Older Adults: A Systematic Mixed Studies Review. Gerontologist.

[68] Sharkiya S.H., Hag A.M. (2024). Environmental and contextual factors influencing e-health use among older adults: A rapid review. Int. J. Med. Inform..

[69] Hunter I., Lockhart C., Rao V., Tootell B., Wong S. (2022). Enabling Rural Telehealth for Older Adults in Underserved Rural Communities: Focus Group Study. JMIR Form. Res..

[70] Choi H., Lee S.-K. (2022). Failure mode and effects analysis of telehealth service of minority elderly for sustainable digital transformation. Comput. Biol. Med..

[71] Zhao J., Wang L., Guo K. (2022). Impact of smart health systems on the behavior of older adults under community healthcare. Front. Public Health.

